# Mast Cells Mediate Inflammatory Injury and Aggravate Neurological Impairment in Experimental Subarachnoid Hemorrhage Through Microglial PAR-2 Pathway

**DOI:** 10.3389/fncel.2021.710481

**Published:** 2021-09-27

**Authors:** Bing Qin, Yucong Peng, Chen Zhong, Yong Cai, Shengjun Zhou, Huaijun Chen, Jianfeng Zhuang, Hanhai Zeng, Chaoran Xu, Hangzhe Xu, Jianru Li, Guangyu Ying, Chi Gu, Gao Chen, Lin Wang

**Affiliations:** Department of Neurosurgery, The Second Affiliated Hospital of Zhejiang University School of Medicine, Hangzhou, China

**Keywords:** subarachnoid hemorrhage, mast cells, neuroinflammation, microglia, PAR-2

## Abstract

Subarachnoid hemorrhage (SAH) is a devastating cerebrovascular disease with high mortality and disability. Aberrant neuroinflammation has been identified as a critical factor accounting for the poor prognosis of SAH patients. Mast cells (MCs), the sentinel cells of the immune system, play a critical in the early immune reactions and participate in multiple pathophysiological process. However, the exact role of MCs on the pathophysiological process after SAH has not been fully understood. The current study was conducted to determine the role of MCs and MC stabilization in the context of SAH. Mouse SAH model was established by endovascular perforation process. Mice received saline or cromolyn (MC stabilizer) or compound 48/80 (MCs degranulator). Post-SAH evaluation included neurobehavioral test, western blot, immunofluorescence, and toluidine blue staining. We demonstrated that SAH induced MCs activation/degranulation. Administration of MC stabilizer cromolyn conferred a better neurologic outcome and decreased brain edema when compared with SAH+vehicle group. Furthermore, cromolyn significantly inhibited neuroinflammatory response and alleviated neuronal damage after SAH. However, pharmacological activation of MCs with compound 48/80 dramatically aggravated SAH-induced brain injury and exacerbated neurologic outcomes. Notably, pharmacological inhibition of microglial PAR-2 significantly reversed MCs-induced inflammatory response and neurological impairment. Additionally, the effect of MCs-derived tryptase in mediating neuroinflammation was also abolished by the microglial PAR-2 blockage *in vitro*. Taken together, MCs yielded inflammatory injury through activating microglia-related neuroinflammation after SAH. These data shed light on the notion that MCs might be a novel and promising therapeutic target for SAH.

## Introduction

Subarachnoid hemorrhage (SAH), mainly caused by ruptured intracranial aneurysm, is a serious cerebrovascular disease with high mortality and chronic disability worldwide (Macdonald and Schweizer, 2017; Hostettler et al., 2020). Although studies on SAH have been carried out for decades, the prognosis of patients with SAH remains unsatisfactory (Rautalin et al., 2020; Savarraj et al., 2020). Among the multiple pathological events that occur after SAH, prolonged inflammation has been identified to be the critical factor accounting for the poor prognosis of SAH. Accumulating studies, including ours, suggested that inhibition of inflammatory response affords a robust neuroprotection against SAH (Gris et al., 2019; Khey et al., 2019; Peng et al., 2020). Thus, it is important to explore the pathophysiological process of neuroinflammation and further develop novel therapeutic strategies to improve the outcome of SAH.

Mast cells (MCs), one of the granulocytes derived from the myeloid stem cell that widely distributed in tissues surrounding blood vessels, nerves, smooth muscle cells, and synovial membranes, are an important regulator participating in both innate immunity and adaptive immunity (El Ansari et al., 2020). Upon activation, MCs could rapidly migrate to the site of injury and undergo degranulation by releasing the preformed mediators, including tryptase, chymase, histamine, heparin, serotonin, and prostaglandins into extracellular, which further recruit immune cells and amplify the inflammatory response (Chelombitko et al., 2020). For years, MCs have been mainly studied due to their pathogenic role in allergic responses during the progression of urticaria and asthma (Falduto et al., 2020). However, recent studies revealed the critical effect of MCs in central nervous system (CNS) disease. In the pathological process of ischemic stroke and acute stress, MCs are believed to be the “first responder” and contribute to blood-brain barrier (BBB) disruption via mediating glial dysfunction (Ribatti, 2015; D’amico et al., 2020). In addition, MCs are associated with the progression of several neurodegenerative and neuropsychiatric diseases, including amyotrophic lateral sclerosis, frontotemporal dementia, and multiple sclerosis (Brown and Weinberg, 2018; Novellino et al., 2020). Furthermore, MCs were recently reported to aggravate the neurological impairment after hemorrhagic stroke (Kuwabara et al., 2017; Furukawa et al., 2020; Zhang et al., 2021). Although the pathophysiological role of MCs in human diseases has been studied for years, little is known about the role of MCs in the pathological process after SAH.

Therefore, the current study was designed to investigate the role of MCs under SAH condition and explore the potential mechanism. Here, for the first time, we reported that MCs contribute to the inflammatory injury after SAH through interacting with microglia in a mouse model of SAH.

## Materials and Methods

### Animals and Subarachnoid Hemorrhage Model

Male C57BL/6J mice (body weight 20–25 g, purchased from Slac Laboratory Animal Co., Ltd., Shanghai, China) were kept at room temperature (22 ± 1°C) with a 12 h day/night cycle (humidity: 60 ± 5%). Mice are free to water and food. All procedures involving animals conformed to the Guide for the Care and Use of Laboratory Animals of the National Institutes of Health and were approved by the Institutional Animal Care and Use Committee of Zhejiang University.

The SAH model was established via endovascular perforation as previously described (Nishikawa et al., 2018). Briefly, mice were anesthetized with 1% pentobarbital (50 mg/kg, i.p.). Subsequently, the left carotid artery and its branches were exposed, and a sharped nylon suture was inserted from the external carotid artery and further went along into the internal carotid artery, and ultimately perforated the bifurcation of the anterior and middle cerebral arteries. Mice in sham group underwent the same procedure except the suture was withdrawn without puncture.

### Study Design

This study was completed in three separate experiments. A total of 234 mice, including the dead ones, was used in the current study as shown in Supplementary Table 1.

#### Experiment 1

To determine the activation of MCs after SAH, mice were randomly divided into sham group and SAH groups with different time points (6, 12, 24, 48, and 72 h). The ipsilateral cerebral cortex was harvested for western blotting and immunofluorescence staining.

#### Experiment 2

To determine the role of MCs in the pathological process of SAH following, the selective MCs stabilizer cromolyn and MCs degranulator compound 48/80 (C48/80) was used. Mice were randomly divided into sham group, SAH + vehicle group, SAH + cromolyn group, and SAH + C48/80 group. Toluidine blue stain, neurological test, brain water content, western blot, and immunofluorescence staining was performed at 24 h post-modeling.

#### Experiment 3

To determine the mechanism of MCs-mediated brain injury, mice were randomly divided into SAH + vehicle group, SAH + C48/80 group, SAH + ENMD-1068 (PAR-2 antagonist) group, and SAH + C48/80 + ENMD-1068 group. Neurological test, brain water content, western blot, and immunofluorescence staining were assessed at 24 h after SAH.

### Drug Administration

Mast cell stabilizer cromolyn (purchased from Med-ChemExpress, NJ, United States), MCs degranulator compound 48/80 (purchased from Cayman Chemicals, MI, United States), and PAR2 antagonist ENMD-1068 (purchased from Med-ChemExpress, NJ, United States) were dissolved in saline with a final concentration of 100 mg/kg, 0.5 mg/mL, and 0.5 mg/kg, separately. Mice received 0.2 ml cromolyn or compound 48/80 intravenously at 5 min before the induction of SAH, while ENMD-1068 was administrated intravenously at 1 h before modeling. The deliver route and concentration of agents were obtained as previously reported (Strbian et al., 2007; de Almeida et al., 2020). Animals in the sham-operated group and SAH + vehicle group received the same volume of saline.

### Subarachnoid Hemorrhage Grade

The severity of SAH was evaluated according to the SAH grading scale as previously reported (Sugawara et al., 2008). Briefly, the basal cistern was divided into six segments, and each segment was scored from 0 to 3 based on the amount of bleeding. A total score ranging from 0 to 18 was obtained by adding the scores together. All the tests and SAH grades were evaluated by an independent researcher.

### Neurological Behavioral Analysis

Two neurological tests were introduced at 24 h after modeling as previously reported (Matsumura et al., 2019; Peng et al., 2020). The modified Garcia scoring system consists of six tests: spontaneous activity, symmetry in limb movement, forepaw outstretching, climbing, body proprioception, and the response to vibrissae touch. Each test was scored as either 0–3 or 1–3, and the total scores ranged from 3 to 18. A higher indicated a better neurological function. Another neurological test was conducted through three activity examinations of appetite, activity, and deficits. Grading of neurologic deficits was as follows: severe neurologic deficit (score = 4–6), moderate neurologic deficit (score = 2–3), mild neurologic deficit (score = 1), and no neurologic deficit (score = 0). A lower score indicated a better neurological function. A higher score suggested a worse neurological deficit. All the neurological performance was evaluated by a blinded investigator.

### Brain Water Content

Brain water content was tested to evaluate the severity of brain edema. Mice were sacrificed at 24 h after modeling and left hemispheres were immediately collected and weighed to get the wet weight. Then, the samples were dried at 105°C for 72 h to get the dry weight. The brain water content was calculated as [(wet weight − dry weight)/(wet weight)] ^∗^100%.

### Western Blot

Western blot was performed as previously described (Xu et al., 2017). Briefly, mice were sacrificed and left hemispheres were harvested. Equal amounts of protein (50 μg) were loaded onto sodium dodecyl sulfate–polyacrylamide gels and electrophoresed. Subsequently, the proteins were transferred to polyvinylidene fluoride (PVDF) membranes. Membranes were incubated overnight at 4°C with primary antibodies: TNF-α (1:5000, Abcam ab6671), IL-1β (1:2000, Santa Cruz SC-23459), IL-6 (1:1000, Santa Cruz SC-1265), PAR-2 (1:500, Abcam, ab180953), and anti-β-actin (1:5000, Proteintech, Cat. No. 60008-1-Ig). The membranes were processed with horseradish-peroxidase conjugated secondary antibodies at room temperature for 1 h. Bands were visualized using the ECL Plus chemiluminescence reagent kit (Millipore, MA, United States). The band densities were quantified with the Image J software (NIH). The results were expressed as the relative density of the band as compared β-actin.

### Brain Tissue Sections

Mice were anesthetized and transcardially perfused with 0.1M PBS followed by 4% paraformaldehyde (pH 7.4). Then, brains were immersed in 4% formaldehyde and then dehydrated in 30% sucrose solution. Coronal frozen sections (8 μm) were placed onto slides for toluidine blue staining and immunofluorescence staining.

### Toluidine Blue Staining

In order to evaluate mast cell activation and degranulation, brain tissue sections were stained with toluidine blue solution (purchased from Bioss biotechnology Co., Ltd, Beijing, China) according to manufacturer’s protocol. The mast cell count was performed on each slide through a microscope by a blinded investigator.

### Immunofluorescence Staining

Brain slices were washed with 0.1M PBS and incubated with 5% BSA containing 0.3% Triton X-100 for 1 h at room temperature. Then, sections were incubated overnight at 4°C with primary antibodies: Iba-1 (1:500, Abcam, ab5076), NeuN (1:500, Abcam, ab104224), PAR-2 (1:500, Abcam, ab180953). After undergoing washes with PBS, the sections were incubated with secondary antibodies for 2 h at 4°C in the dark. Terminal deoxynucleotide transferasedeoxyuridine triphosphate (dUTP) nick end labeling (TUNEL) staining was performed to detect apoptotic cell death according to the manufacturer’s protocol (Roche Inc., Basel, Switzerland). Each mouse had 4 brain slides examined, and each slide was examined under three fields of vision to acquire the mean number of target cells using a fluorescence microscope (Olympus, Tokyo, Japan) by a blinded investigator. The data were expressed as cells per square millimeter.

### Microglia Culture and Quantitative Real-Time Polymerase Chain Reaction

Murine BV2 cells were cultured in the DEME medium with 10% fetal bovine serum and 1% penicillin/streptomycin (Gibco, United States) under the atmosphere of 5% carbon dioxide at 37°C. A total of 250 nM recombinant mouse tryptase (synthesize by GL Biochem [Shanghai] Ltd, China) was introduced into the medium for 6 h to simulate MCs degranulation insult *in vitro* as previously described. After incubation, the medium was removed and BV2 cells were collected for Quantitative Real-Time PCR (RT-PCR) assay. To further determine the potential molecular mechanism, BV2 cells were pretreated with 500 μM PAR-2 antagonist ENMD-1068 (purchased from Med-ChemExpress, NJ, United States) for 4 h before tryptase incubation. The administration of tryptase and ENMD-1068 were obtained base on the previous study (Zhang et al., 2019).

Total RNA of cultured BV2 cell was extracted from using TRIzol reagent (Invitrogen, United States). RNA was reverse transcribed into cDNA with a RevertAid First Strand cDNA Synthesis Kit (Thermo Fisher Scientific, United States). The RT-PCR was conducted with UltraSYBR Mixture (CWBio, China), specific primers (as shown in Supplementary Table 2), and cDNA on a Mx3000P real-time PCR system (Agilent Technologies, United States). GAPDH was set as internal control, and results were presented as fold changes compared with the control group.

### Statistical Analysis

The data were shown as the mean ± standard deviation (SD). Differences among the groups were analyzed using one-way analysis of variance (ANOVA) followed by Tukey’s multiple comparison test. All statistical analyses were performed on SPSS (version 22.0), and statistical significance was determined as *P* < 0.05.

## Results

### Mast Cells Rapidly Actives in the Brain After Subarachnoid Hemorrhage Insult

**FIGURE 1:**
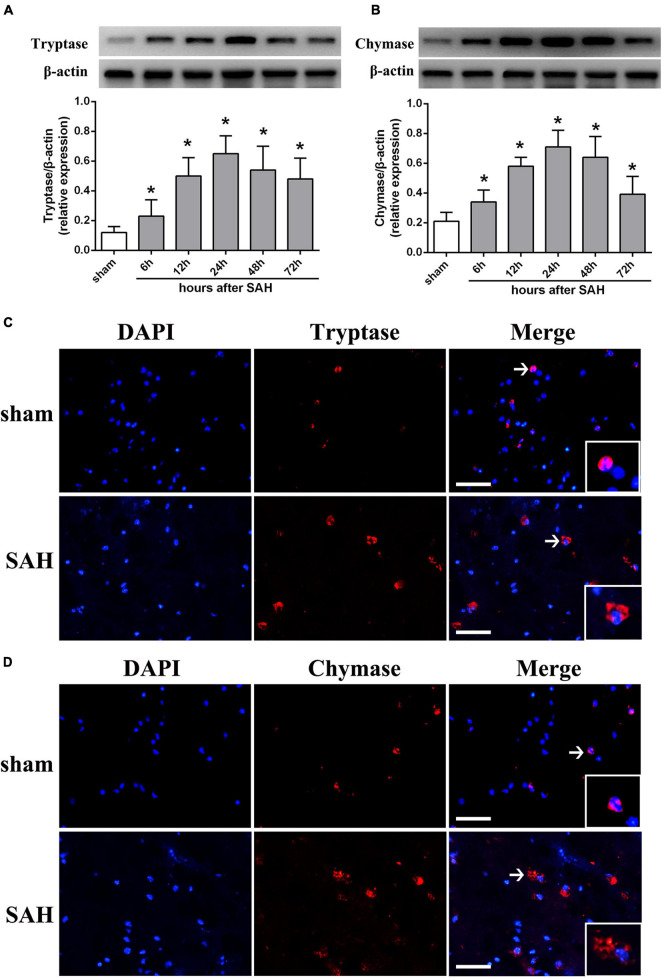
The expression pattern of MCs-derived tryptase and chymase after SAH. **(A,B)** Representative western blotting images and quantitative analyses of tryptase and chymase expression in ipsilateral basal cortex after SAH. *n* = 6. The bars represent the mean ± SD. *P < 0.05 versus sham. **(C,D)** Representative microphotographs of immunofluorescence double staining showing the expression and distribution of tryptase and chymase in sham group and SAH 24 h group. Scale bar = 50 μm.

To determine whether MCs could response to SAH in brain, western blot was performed to evaluate the expression of tryptase and chymase, two kinds of critical proteases generated by MCs after activation. The result indicated that the expression of tryptase significantly increased at 6 h and peaked at 24 h after SAH (Figure 1A). A similar tendency was observed in the expression of chymase, which started to increase at 6 h and peaked at 24 h after SAH (Figure 1B). Additionally, immunostaining further confirmed the increased expression of tryptase and chymase post-modeling. More importantly, both tryptase and chymase demonstrated a tendency of degranulation with spread from intracellular to extracellular after SAH (Figures 1C,D).

### Mast Cells Activation Aggravated Brain Edema and Neurological Deficits After Subarachnoid Hemorrhage

**FIGURE 2:**
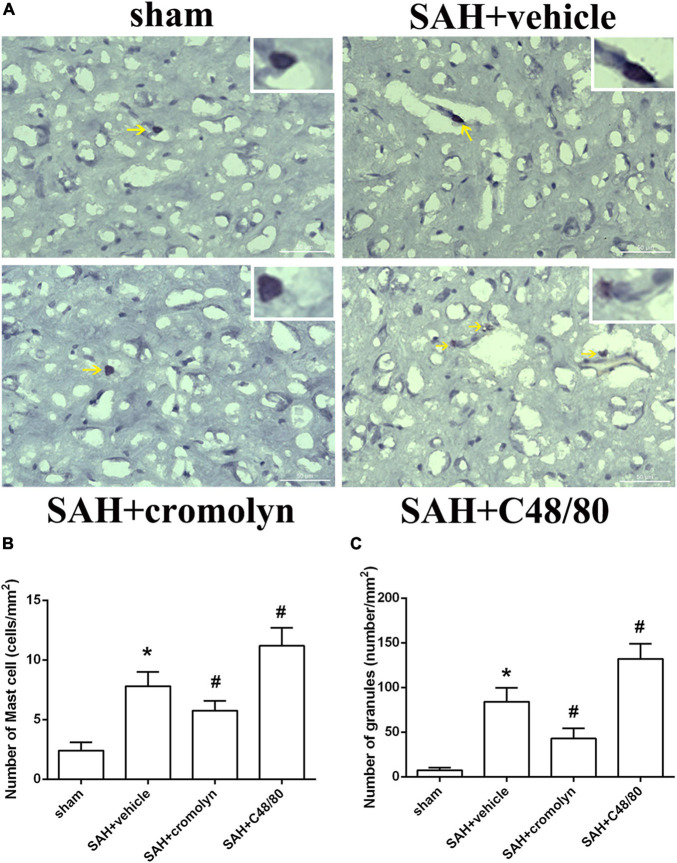
Effect of cromolyn and compound 48/80 on MCs infiltration and degranulation at 24 h after SAH. **(A)** Toluidine blue staining of mast cells in cortex region (yellow arrow: infiltrated MCs). **(B,C)** Quantitative analyses of MCs infiltration and degranulation. *n* = 6. Data are represented as mean ± SD. *P < 0.05 versus sham group. #*P* < 0.05 versus SAH + vehicle group.

To investigate the effect of MCs on the pathological process following SAH, the selective MCs stabilizer cromolyn and MCs degranulator compound 48/80 were introduced. Toluidine blue staining suggested that there was an increased MCs infiltration and degranulation after SAH compared to sham group (Figure 2), which was consistent with increased level of tryptase and chymase (as shown in Figure 1). Furthermore, when compared with SAH + vehicle group, the MCs infiltration and degranulation was significantly inhibited by the administration of cromolyn, while compound 48/80 remarkably promoted MCs activation (Figure 2), suggesting the efficacy of the mentioned stabilizer and degranulator in brain under SAH condition.

**FIGURE 3:**
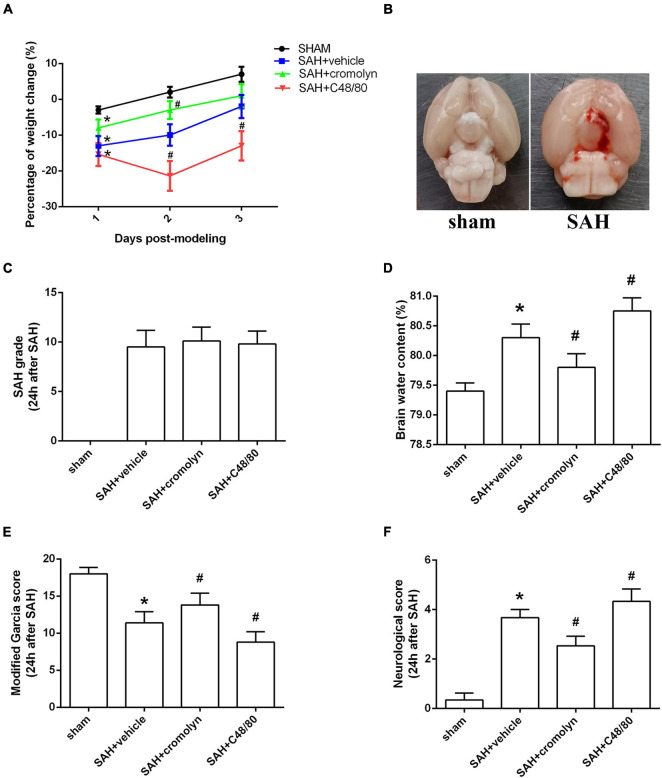
Effect of MCs on brain edema and neurological outcome at 24 h post-modeling. **(A)** The change of body weight in animals among different groups. **(B)** Typical brains from sham-operated and SAH mice. **(C)** The quantification of SAH severity. **(D)** The quantification of brain water content. **(E,F)** The quantification of neurobehavioral test. *n* = 6. Data are represented as mean ± SD. *P < 0.05 versus sham group. #*P* < 0.05 versus SAH + vehicle group.

There was a significant loss of body weight in modeling groups. And compared with SAH + vehicle group, mice in SAH + cromolyn group demonstrated a faster weight gain after SAH (Figure 3A). When the animals were sacrificed, subarachnoid blood clots were observed around the circle of Willis and the ventral brainstem. No significant difference in SAH grade among modeling groups was noted (Figures 3B,C). Moreover, remarkable neurobehavioral dysfunction and increased brain water content were observed in the modeling groups when compared with the sham group. Post-SAH treatment with MCs stabilizer cromolyn significantly decreased brain water content and alleviated neurological deficits, while mice administrated with MCs degranulator compound 48/80 suffered a worse brain edema and neurological impairment (Figures 3D–F).

### Mast Cells Mediated Neuroinflammation and Microglial Activation

**FIGURE 4:**
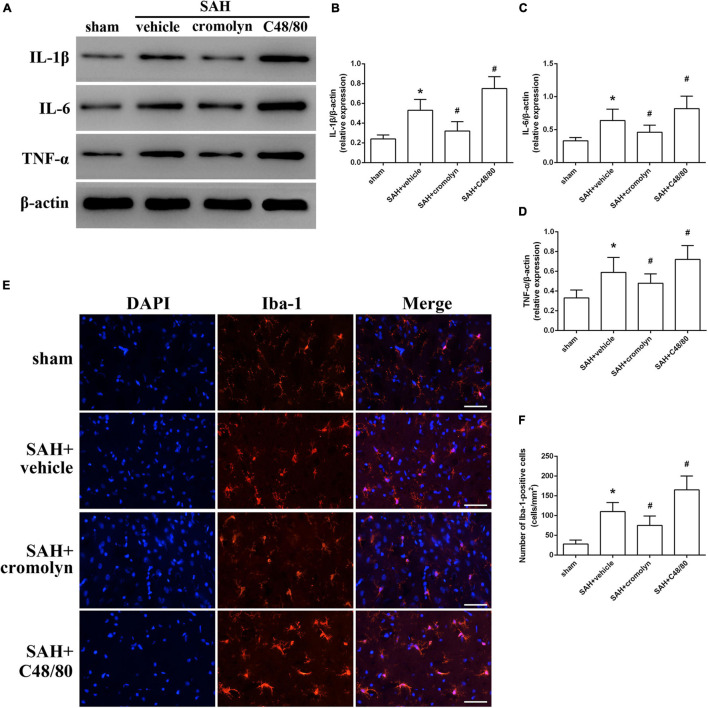
Effect of MCs on SAH-induced neuroinflammation at 24 h post-modeling. **(A–D)** Representative picture and quantitative analysis of expression of TNF-α, IL-1β, and IL-6 in different groups. **(E,F)** Representative picture and quantitative analysis of microglia activation in ipsilateral basal cortex after SAH. scale bar = 50 μm. *n* = 6. Data are represented as mean ± SD. *P < 0.05 versus sham group. #*P* < 0.05 versus SAH + vehicle group.

Given the critical role of inflammatory response on determining the outcome of SAH, the next part was conducted to determine the adverse effects of MCs in the pathological process following SAH were exerted through mediating neuroinflammation. Data from western blot indicated that the expression of pro-inflammatory mediators, including TNF-α, IL-1β, and IL-6, were significantly increased after SAH (Figures 4A–D). And treatment with cromolyn significantly downregulated the expression of the mentioned cytokines, while compound 48/80 administration further upregulated the level of these cytokines (Figures 4A–D). Accordingly, the number of microglia, one of the most important immune cells in mediating neuroinflammation, was increased after SAH (Figures 4E,F). Compared with SAH + vehicle group, cromolyn treatment significantly decreased the activation of microglia, while MCs degranulator compound 48/80 further promoted the microglial activation (Figures 4E,F).

### Mast Cells Activation Mediated Neuronal Injury After Subarachnoid Hemorrhage

**FIGURE 5:**
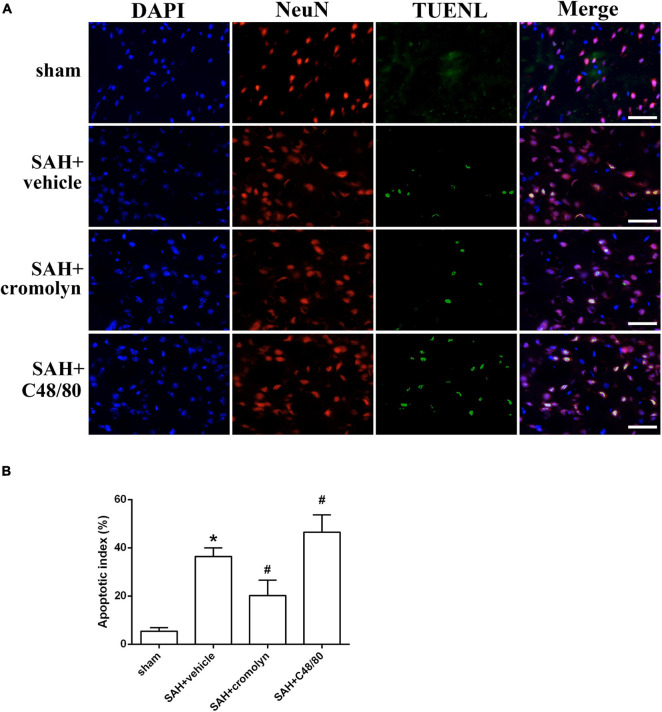
Effect of MCs on SAH-induced neuronal injury. **(A)** Representative microphotographs showing the co-localization of TUNEL positive cells (green) with NeuN (red) in cortex. **(B)** The quantitative analysis of TUNEL-positive neurons. Scale bar = 50 μm. *n* = 6. Data are represented as mean ± SD. *P < 0.05 versus sham group. #*P* < 0.05 versus SAH + vehicle group.

TUNEL staining was conducted to evaluate the effect of MCs on neuronal injury. As shown in Figure 5, the percentage of TUNEL-positive neurons significantly increased in SAH groups when compared with sham group, suggesting a significant neuronal injury after SAH (Figure 5). And treatment with cromolyn significantly reversed these processes, while administration of compound 48/80 further upregulated the percentage of TUNEL-positive neurons (Figure 5).

### Blockade of Microglial Protease-Activated Receptor-2 Alleviated Mast Cells-Mediated Inflammatory Injury

**FIGURE 6:**
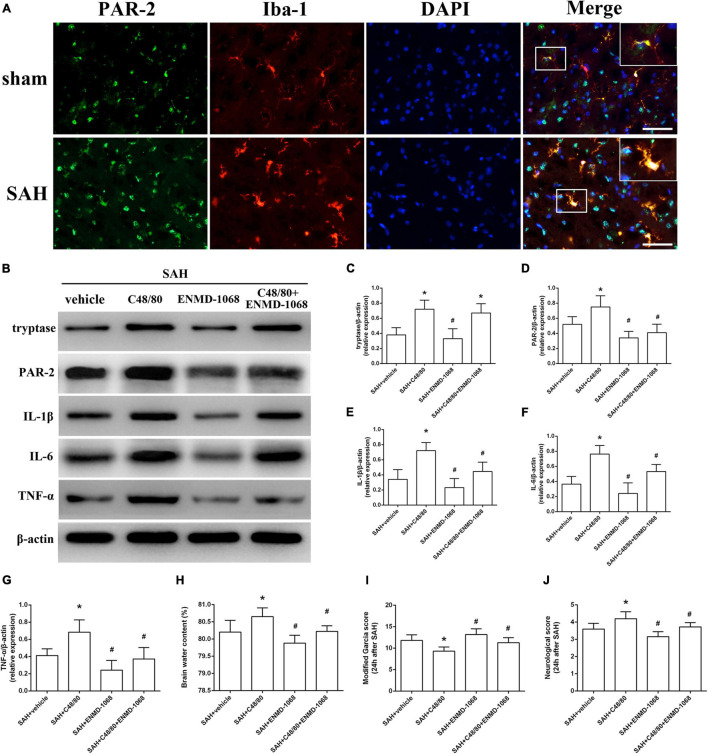
Pharmacological inhibition of microglial PAR-2 alleviated MCs-mediated inflammatory injury at 24 h after SAH. **(A)** Immunofluorescence staining demonstrates the expression of PAR-2 in sham and SAH mice. *n* = 6. Scale bar = 50 μm. **(B–G)** Representative western blotting images and quantitative analyses of tryptase, PAR-2, TNF-α, IL-1β, and IL-6 expression in ipsilateral basal cortex after SAH. **(H)** The quantification of brain water content. **(I,J)** The quantification of neurobehavioral test. *n* = 6. Data are represented as mean ± SD. *P < 0.05 versus SAH + vehicle group. #*P* < 0.05 versus SAH + compound 48/80 group.

Protease-activated receptor-2 (PAR-2) is a MC-tryptase receptor located in microglia. Consistent with the increased expression of tryptase, immunostaining and western blot demonstrated an upregulated level of PAR-2 after SAH (Figures 6A,B). To further investigate the mechanism of MCs-mediated neuroinflammation under SAH condition, the selective PAR-2 antagonist ENMD-1068 was introduced. Compared with SAH + vehicle group, MCs degranulator compound 48/80 conferred a robust inflammatory response, which was evidenced by the increased expression of tryptase, PAR-2, TNF-α, IL-1β, and IL-6. However, although it did not affect the level of tryptase, PAR-2 antagonist ENMD-1068 significantly reversed the pro-inflammatory effect of compound 48/80, evidenced by decreased level of inflammatory cytokines (Figures 6B–G). More importantly, the adverse effects of compound 48/80 on aggravating brain edema and neurological impairment were significantly reversed by ENMD-1068 (Figures 6H–J).

### Effect and Mechanism Studies *in vitro*

**FIGURE 7:**
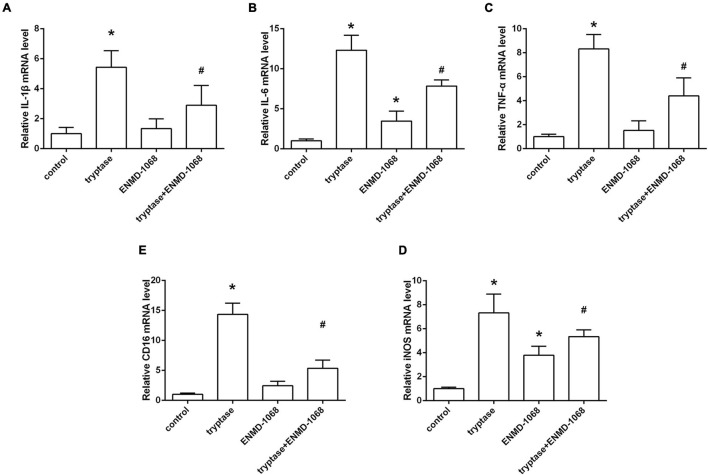
Effect and mechanism studies using cultured BV2 cell *in vitro*. **(A–E)** Quantification of RT-PCR on the relative mRNA level of pro-inflammatory cytokines genes (IL-1β, IL-6, and TNF-α) and M1 microglia markers genes (CD16, iNOS). Data were represented as mean ± SD. *P < 0.05 versus control group. #*P* < 0.05 versus tryptase group.

The cultured BV2 cells were incubated with mast cell derived tryptase and the level of inflammatory cytokines was analyzed by RT-PCR. Consistent with the results *in vivo*, data from RT-PCR indicated that stimulate microglia with mast cell derived tryptase significantly increased the level of TNF-α, IL-1β, and IL-6, accompanied by the upregulation of microglial M1 marker CD16 and iNOS (Figure 7). More importantly, blockage of microglial PAR-2 with ENMD-1068 remarkably alleviated tryptase-induced upregulation of mentioned inflammatory mediators and microglial M1 markers (Figure 7), suggesting that mast cell-derived tryptase promoted microglia polarized into pro-inflammatory M1 phenotype and aggravated microglia-associated neuroinflammation, at least partly, by interacting with microglial PAR-2.

## Discussion

**FIGURE 8:**
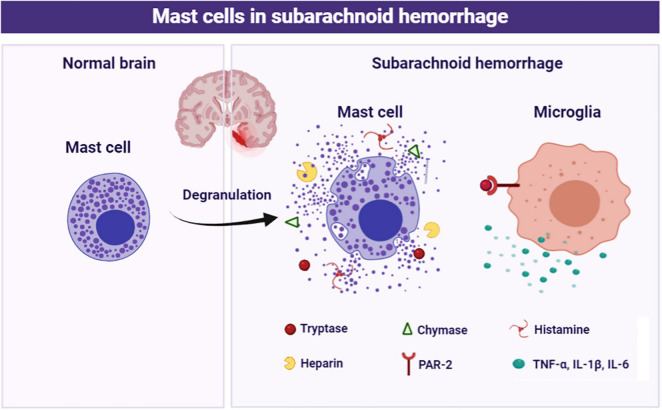
Schematic diagram of MCs in the pathological process following SAH. MCs are rapidly activated and degranulation in response to SAH. Subsequently, MCs-derived tryptase binds to PAR-2 on microglia, resulting in microglial activation and ultimately mediate inflammatory damage.

In the current study, we focused on the role of brain MCs in the pathological process following SAH and explored the potential mechanism. The major findings of the study are listed as follows: (1) MCs rapidly activated in response to SAH; (2) MCs stabilizer cromolyn significantly alleviated SAH-induced brain edema and neurological deficits, as well as attenuated neuroinflammation and neuronal damage, while MCs degranulator compound 48/80 exacerbated brain injury following SAH; (3) Pharmacological inhibition of microglial PAR-2 reversed MCs-mediated inflammatory response and neurological impairment after SAH. Based on the evidence above, MCs contribute to brain injury through mediating microglia-related neuroinflammation under SAH condition (Figure 8).

Subarachnoid hemorrhage accounts for 5% of all the stroke cases and mortality could be over 67% in the first few months. And the survivors could suffer from chronic neurological disorders, such as cognitive and/or motor impairments (Muehlschlegel, 2018). Although the pathophysiological event that occurs after SAH is quite complex, recent studies increasingly implicate that excessive inflammation is the critical factor in determining the prognosis of SAH (Lucke-Wold et al., 2016). Neuroinflammation is a complex mechanism involving different immune and inflammatory cells and different inflammatory mediators. Previous studies indicated that inflammatory response peaks at 24–48 h post-bleeding. And the elevated neuroinflammation is associated with higher clinical severity, delayed cerebral ischemia, mortality, and functional outcome after SAH (Ahn et al., 2019; Bevers et al., 2019). In addition, recent studies suggested that inhibition of Enhancer of Zeste Homolog 2 (EZH2) or thromboxane-prostaglandin (TP) receptors could afford a robust neuroprotective effect against SAH via inhibiting neuroinflammation in a rat model of SAH (Lagier et al., 2019; Luo et al., 2020). Similarly, our previous studies have also confirmed that inhibition of neuroinflammation confers a robust neuroprotection against SAH in murine models (Xu et al., 2017; Peng et al., 2020). Based on the evidence above, further investigations to discover new therapeutic strategies targeting SAH-induced neuroinflammation remain a high priority.

Mast cells are the immune cell widely located in regions in contact with the external environment, including respiratory tract, skin, and gut (Metz et al., 2007). Due to the peculiar anatomical location, MCs act as the first line immune sentinel cells in responding to allergens, pathogen, or environmental stimulation such as cold. Once activated, MCs rapidly respond to signals from the surrounding microenvironment and attendant pathological conditions via migrating to an injury site and further mediating tissue damage and/or repair through releasing the granule into surroundings (El Ansari et al., 2020). On the one hand, the activated MCs could facilitate wound healing by recognizing antigens through pattern recognition receptors and the high-affinity immunoglobulin E receptor, resulting in the releasing of granules, which further mediates cell recruitment, fibrosis, angiogenesis, and extracellular matrix deposition (Ozpinar et al., 2020). One the other hand, the cytoplasmic granules from MCs also contribute to multiple inflammatory and allergic pathogenesis diseases, including asthma, allergy, arthritis, interstitial cystitis, irritable bowel syndrome (IBD), ulcers, and prostatitis (Chelombitko et al., 2020; Fu et al., 2020). Notably, accumulating studies implicate the role of MCs in CNS. Similar to the effects in peripheral tissue, MCs in the brain confer a complex role in maintaining CNS homeostasis, both beneficial and adverse (da Silva et al., 2014). MCs are necessary for the normal neuronal development and cognitive function (Fitzpatrick and Morrow, 2017; Lenz et al., 2018). However, excessive activation of MCs has also been identified to participate in the pathological process of multiple CNS diseases. Liu et al. reported that MCs activation was closed related to the postoperative cognitive dysfunction (POCD) (Liu and Yin, 2018). Similarly, aberrant activation of MCs could mediate neuroinflammation via interaction with glial cells and neurons in Parkinson’s disease (PD) and depression (Kempuraj et al., 2019; Friesen et al., 2020). Moreover, MCs activation significantly exacerbates brain edema and neuronal damage in mice subjected to both middle cerebral artery occlusion (MCAO) and intracerebral hemorrhage (ICH) through degrade neurovascular matrix and open tight junction, while MC-deficient rats demonstrated a reduced mortality, brain swelling, and neurological outcome (McKittrick et al., 2015; Parrella et al., 2019). Moreover, an increased number of infiltrated MCs was reported in the aneurysm tissues of intracranial aneurysm patients, and MCs stabilizer reduced the size and the thinning of aneurysm, suggesting a critical role of MCs in the development of aneurysm (Ishibashi et al., 2010; Hasan et al., 2012). Despite the fact that the activation of MCs has been determined in multiple human diseases, the potential function of MCs in the pathological process after SAH is yet to be elucidated.

Therefore, in the first part of study, we explored whether MCs are involved in the pathological process after SAH. And consistent with previous studies demonstrating the MCs activation at early stage after TBI (Kempuraj et al., 2020), we noted a significant and rapid activation of MCs under SAH, evidenced by the increased expression of cytoplasmic proteases and degranulation of mast cells in the brain. Given the critical roles of MCs in CNS homeostasis and diseases, we speculated that MCs might influence the outcome of SAH. Thus, in the next part of this study, the role of MCs activation after SAH was investigated by using cromolyn and compound 48/80. Cromolyn is one of the most widely used MCs stabilizers in the management of asthma worldwide through preventing MCs degranulation and inhibiting the release of inflammatory mediators such as histamine and tryptase, while compound 48/80 is an agent with a robust effect in modulating MCs degranulation. And consistent with previous study (Strbian et al., 2007), we noted that administration of cromolyn significantly inhibited the activation and degranulation of MCs, while compound 48/80 enhanced the activation of MCs, indicating the efficacy of both compounds in the pathological process following SAH. McKittrick et al. (2015) reported that MCs activator compound 48/80 significantly aggravated cerebral edema and neurological dysfunction in a rat ischemic stroke model via upregulating gelatinase activity, while knockdown of MCs diminished brain swelling, BBB leakage, and neutrophils infiltration. Similarly, in the current study, we noted that inhibition of MCs with cromolyn confers a robust neuroprotection via decreasing brain edema and neurological deficits, while compound 48/80 significantly aggravated SAH-induced brain injury. Moreover, given the critical role of inflammation in determining the outcome of SAH, we further explore the effect of MCs in SAH-induced neuroinflammation. As reported (Hughes et al., 2017), our data indicated that MCs stabilizer remarkably inhibited inflammatory response, evidenced by the decreased number of activated microglia and downregulated pro-inflammatory cytokines. Additionally, treatment with cromolyn significantly alleviated neuronal damage. However, activation MCs with compound 48/80 further deteriorate SAH-induced inflammation and neuronal injury. All of this evidence suggested that activation of MCs affords to brain damage after SAH via mediating inflammatory injury. However, the potential mechanism remains unclear.

Therefore, the last part of this study was aimed to determine the exact mechanism of MCs-related neuroinflammation under SAH condition. Microglia are the most important resident immune cell distributed in the CNS. Generally, microglia keep in rest status, morphology characterized by a small soma and long protrusions, and provide immune surveillance for injury and pathogen. Upon activation, microglia could rapidly migrate to the site of injury and transform into different phenotypes with distinct physiological functions. Among the multiple phenotypes, M1 microglia, characterized by the CD16 and iNOS, could secrete pro-inflammatory cytokines and chemokines (such as IL-1β and TNFα), ultimately elevating the immune response and exacerbating brain damage at the early stage after SAH (Xiong et al., 2016). Accumulating evidence indicated that inhibition of the microglia-related inflammation conferred a robust neuroprotective effect against SAH (Xie et al., 2018; Gris et al., 2019; Peng et al., 2020). Notably, recent studies indicated that MCs might be an important factor in modulating microglial activation. On the one hand, MCs degranulation secretes histamine and proteases, which recruit monocytes and neutrophils, resulting in an inflammatory microenvironment and microglial activation (Christy et al., 2013). On the other hand, MCs could activate microglia directly through interacting with numerous receptors distributed on the surface of microglia (Conti et al., 2020; Sandhu and Kulka, 2021). Among the multiple participants, proteinase-activated receptor 2 (PAR-2) is one of the most important MCs-microglial interaction-related receptors. PAR-2, also known as GPR11 or F2RL1, belongs to the 7-transmembrane receptors that couple to guanosine-nucleotide-binding proteins family that are encoded by F2RL1 gene (Kawaguchi et al., 2020). PAR-2 is widely expressed in mammals, especially in epidermal keratinocytes and immune cells, including neutrophils, dendritic cells, T cells, and macrophages (Sutherland et al., 2019; Shah et al., 2020). Upon its extracellular amino terminus between arginine and serine was cleaved by protease, PAR-2 is activated and further modulate multiple biological processes such as metabolism, tumorigenesis, and inflammatory response (Kim et al., 2020; Wang et al., 2020; Bang et al., 2021; Jiang et al., 2021). Recent studies indicated PAR-2 is the critical bridge between the cross-talk of MCs and microglia. Tryptase released from MCs could cleave and activate proteinase-activated receptor 2 (PAR-2) on microglial cells. Subsequently, the activated PAR-2 could further phosphorylate mitogen-activated protein kinases (MAPK), resulting in the activation of microglia and release of inflammatory cytokines (Zhang et al., 2016). Recently, Ocak et al. (2020) reported that microglia PAR-2 signal was significantly activated, accompanied with the enhanced neuroinflammation in a rat model of asphyxial cardiac arrest. Similarly, in the current study, we noted that the level of microglial PAR-2 was remarkably upregulated after SAH, accompanied by the increased level of pro-inflammatory mediators TNF-α, IL-1β, and IL-6. However, pharmacological inhibition of PAR-2 remarkably reversed MCs-mediated inflammatory response, evidenced by the decreased pro-inflammatory mediators expression and downregulation of M1 microglia markers. More important, blockage of PAR-2 significantly diminished MCs-induced brain edema and neurological impairment after SAH. All the mentioned evidence suggested a critical role of microglial PAR-2 in MCs-induced tryptase-dependent neuroinflammation in the pathological process of SAH.

Several limitations of this study should not be ignored. Firstly, we mainly focused on the role of MCs in mediating microglia-related neuroinflammation in the current study. However, given that PAR-2 are widely expressed in the CNS, further studies to determine the potential interaction between MCs with other type of cells, such as astrocyte and endothelial cells, are required in the future. Secondly, both the delivery routes of cromolyn or PAR-2 antagonist ENMD-1068 administrated in this study were obtained from previous studies. It could be important to further explore the pharmacokinetics of these small molecule compounds after SAH *in vivo* and develop novel approaches to further promote translational potential as SAH treatment. Additionally, MCs *per se* can secrete a variety of cytokines, and it is unclear whether the effect of cromolyn or C48/80 on pro-inflammatory cytokines secretion was partly exerted by regulating MCs itself. Lastly, neuroinflammation has been considered as a double-edged sword in the pathophysiological process after acute CNS. Therefore, more efforts might be required to determine the possible effects of MCs at the subacute or chronic stages of SAH.

In summary, the current study investigated the role of MCs in the pathological process after SAH and explored the potential mechanism. Our data indicated that MCs activation contributes to brain edema and neurological impairment after SAH. Furthermore, MCs-derived tryptase exacerbates microglia-related neuroinflammation via interacting with microglial PAR-2. Taken together, the current study supports the notion that targeting MCs or PAR-2 might be a novel and promising therapeutic strategy for SAH.

## Data Availability Statement

The original contributions presented in the study are included in the article/Supplementary Material, further inquiries can be directed to the corresponding author/s.

## Ethics Statement

The animal study was reviewed and approved by the Institutional Animal Care and Use Committee of Zhejiang University. All procedures involving animals conformed to the Guide for the Care and Use of Laboratory Animals of the National Institutes of Health.

## Author Contributions

BQ, SZ, CZ, YC, and HC performed the experiments. JZ, HZ, CX, and HX analyzed the data. YP, JL, GY, CG, and LW wrote and edited the manuscript. LW, YP, and GC designed and supervised the project. All authors contributed to the article and approved the submitted version.

## Conflict of Interest

The authors declare that the research was conducted in the absence of any commercial or financial relationships that could be construed as a potential conflict of interest.

## Publisher’s Note

All claims expressed in this article are solely those of the authors and do not necessarily represent those of their affiliated organizations, or those of the publisher, the editors and the reviewers. Any product that may be evaluated in this article, or claim that may be made by its manufacturer, is not guaranteed or endorsed by the publisher.
