# Supplementary material for: Mast Cells Mediate Inflammatory Injury and Aggravate Neurological Impairment in Experimental Subarachnoid Hemorrhage Through Microglial PAR-2 Pathway
Source: Front Cell Neurosci. 2021 Sep 27;15:710481. doi: 10.3389/fncel.2021.710481 (PMC8503547; doi:10.3389/fncel.2021.710481)
Supplement: Supplementary file 1 [file Table_1.DOCX]

**Supplementary Table 1**

Experimental design and the number of animals used per group.

| **Part** | **Groups** | **Sum** | **Death** | **Neuro and weight test** | **SAH**  **grade** | | **BWC** | **Western blot** | **IF or/and TB staining** |
| --- | --- | --- | --- | --- | --- | --- | --- | --- | --- |
| Ⅰ | sham | 12 | 0 | 0 | 0 | 0 | | 6 | 6 |
|  | SAH 6h | 6 | 0 | 0 | 0 | 0 | | 6 | 6 |
|  | SAH 12h | 6 | 0 | 0 | 0 | 0 | | 6 | 6 |
|  | SAH 24h | 13 | 1 | 0 | 0 | 0 | | 6 | 6 |
|  | SAH 48h | 8 | 2 | 0 | 0 | 0 | | 6 | 6 |
|  | SAH 72h | 7 | 1 | 0 | 0 | 0 | | 6 | 6 |
| Ⅱ | sham | 18 | 0 | 24 | 18 | 6 | | 6 | 6 |
|  | SAH+vehicle | 27 | 3 | 24 | 18 | 6 | | 6 | 6 |
|  | SAH+cromolyn | 27 | 3 | 24 | 18 | 6 | | 6 | 6 |
|  | SAH+C48/80 | 28 | 4 | 24 | 18 | 6 | | 6 | 6 |
| Ⅲ | SAH+vehicle | 21 | 3 | 18 | 18 | 6 | | 6 | 6 |
|  | SAH+C48/80 | 21 | 3 | 18 | 18 | 6 | | 6 | 6 |
|  | SAH+ ENMD-1068 | 20 | 2 | 18 | 18 | 6 | | 6 | 6 |
|  | SAH+C48/80+ENMD-1068 | 20 | 2 | 18 | 18 | 6 | | 6 | 6 |

Neuro test, neurological test; BWC, brain water content; IF staining，immunofluorescence staining;

TB staining, Toluidine blue staining; SAH, subarachnoid hemorrhage.
