# Supplementary material for: Mast Cells Mediate Inflammatory Injury and Aggravate Neurological Impairment in Experimental Subarachnoid Hemorrhage Through Microglial PAR-2 Pathway
Source: Front Cell Neurosci. 2021 Sep 27;15:710481. doi: 10.3389/fncel.2021.710481 (PMC8503547; doi:10.3389/fncel.2021.710481)
Supplement: Supplementary file 2 [file Table_2.DOC]

**Supplementary Table S2.** Information of primers used for RT-PCR

| Gene | Sense Primer (5'-3') | Antisense Primer (3'-5') |
| --- | --- | --- |
| CD16 | ACTGTGGTTGGCTTTTGGGAT | GAGTGATTTCTGACTGGCTGCTG |
| iNOS | AATGCCCGTACCAGGCCCAAT | GGTCACCTACCGCACCCGAGAT |
| IL-1β | TTGTTCATCTCGGAGCCTGTA | AGCACCTTCTTTTCCTTCATC |
| IL-6 | GCACTAGGTTTGCCGAGTAGA | AAGCTGGAGTCACAGAAGGAG |
| TNF-α | ATCCGCGACGTGGAACTG | ACCGCCTGGAGTTCTGGAA |
| GAPDH | AAGAAGGTGGTGAAGCAGG | GAAGGTGGAAGAGTGGGAGT |
